# Lateralization Value of Low Frequency Band Beamformer Magnetoencephalography Source Imaging in Temporal Lobe Epilepsy

**DOI:** 10.3389/fneur.2018.00829

**Published:** 2018-10-05

**Authors:** Yicong Lin, Zhiguo Zhang, Xiating Zhang, Yingxue Yang, Zhaoyang Huang, Yu Zhu, Liping Li, Ningning Hu, Junpeng Zhang, Yuping Wang

**Affiliations:** ^1^Department of Neurology, Xuanwu Hospital, Capital Medical University, Beijing, China; ^2^Beijing Key Laboratory of Neuromodulation, Beijing, China; ^3^School of Biomedical Engineering, Health Science Center, Shenzhen University, Shenzhen, China; ^4^Department of Medical Information Engineering, Sichuan University, Chengdu, China; ^5^Center of Epilepsy, Beijing Institute for Brain Disorders, Capital Medical University, Beijing, China

**Keywords:** magnetoencephalography, beamformer, single equivalent current dipole, temporal lobe epilepsy, low frequency band

## Abstract

**Objective:** In presurgical evaluation of temporal lobe epilepsy (TLE), selection of the resection side is challenging when bilateral temporal epileptiform discharges or structural abnormalities are present. We aim to evaluate the lateralization value of beamformer analysis of magnetoencephalography (MEG) in TLE.

**Methods:** MEG data from 14 TLE patients were analyzed through beamformer analysis. We measured the hemispherical power distribution of beamformer sources and calculated the lateralization index (LI). We calculated the LI at multiple frequencies to explore the frequency dependency and at the delta frequency to define laterality. LI values ranging from −1 to −0.05 indicated right hemispheric dominance. LI values ranging from 0.05 to 1 indicated left hemispheric dominance. LI values ranging from −0.05 to 0.05 defined bilaterality. We measured the power of beamformer sources with a 9-s duration to explore time dependency.

**Results:** The beamformer analysis showed that 10/14 patients had power dominance ipsilateral to resection. The delta frequency band had a higher lateralization value than other frequency bands. A time-dependent power fluctuation was found in the delta frequency band.

**Conclusions:** MEG beamformer analysis, especially in the delta band, might efficiently provide additional information regarding lateralization in TLE.

## Introduction

In presurgical strategies for drug-resistant epilepsy, accurate lateralization is fundamental for temporal lobe epilepsy (TLE) surgery. However, for some cases, successful lateralization using scalp video electroencephalogram (EEG) alone is difficult, especially when bilateral temporal epileptiform discharges are present, regardless of the degree of preponderance to one side (1). In TLE, 35–61% of patients had bilateral temporal independent interictal epileptiform discharges, and 9% of unilateral spike patients who were seizure-free showed only contralateral interictal epileptiform discharges (2–7). If surgery is pursued in these patients, selection of the resection side is extremely challenging. At most epilepsy centers, epileptologists will proceed with intracranial EEG monitoring to aid lateralization. However, a good surgery outcome is not necessarily achieved even when intracranial EEG can lateralize recorded seizures to one side (8). Epileptologists are seeking other non-invasive techniques (9) or a combination of multiple techniques (10) to aid lateralization. For example, EEG and magnetoencephalography (MEG) yield confirmatory and complementary information; therefore, the combination of these two techniques could provide more comprehensive information compared with either technique applied alone in mesial TLE (11).

MEG, as a mature non-invasive tool, has some distinct advantages and is playing a growing role in presurgical strategy. Because the effects of complex layering of head tissues can be largely ignored, source modeling is more straightforward for MEG than EEG, in which an accurate head model needs to be generated that includes the distribution of electrical conductivities of head tissues (12–14). MEG measures are reference-free, unlike EEG, whose analysis considers the location of the reference electrode. Localization accuracy is one of the major advantages of MEG. The maximum MEG localization bias is ~1 cm, while localization errors of up to 25 mm are produced by EEG source modeling under the same experimental conditions (15). In epilepsy cases, MEG appears to have benefits over EEG for the investigation of extratemporal epilepsies, while the superiority and additional value of MEG accuracy in the sublobar range in temporal epilepsy remains unclear (16). Many MEG source localization algorithms have been developed over the past decades to predict the locations of epileptogenic regions (17). The single equivalent current dipole (SECD) model is the most commonly used model in clinical applications. SECD can provide excellent epileptogenic source location when MEG waveforms are very focal, but it relies on the presence of epileptiform discharges (18). Other source localization methods, including beamformer, low-resolution brain electromagnetic tomography (LORETA), standard LORETA, multiple signal classification (MUSIC), and dynamic statistical parametric maps (dSPM), have also been used in epilepsy studies (19). Among them, beamformer, a class of spatial filters, has attracted much attention. Beamformer passes the desired signal at each testing source location while blocking signals from other locations. Beamformer can accurately extract each source waveform within brain volumes, thus enabling further analysis.

In the present study, we aim to test MEG beamformer analysis in temporal epilepsy cases, anticipating that this method may provide lateralization information. We retrospectively analyzed the MEG data of 14 TLE patients using beamformer and compared lateralization results with resection sides. To exclude various factors that might lead to seizure recurrence, such as incomplete surgical resection and inappropriate tapering of antiepileptic drugs (AEDs), non-seizure-free cases were excluded. The presence of these factors would hinder the evaluation of the true value of beamformer in lateralization.

## Materials and methods

### Subjects

We identified patients by reviewing the epilepsy surgical database over a 3-year period (September 2009 to February 2012). All of these cases were discussed by epileptologists together during an epilepsy center presurgical conference in our hospital. We enrolled 14 representative patients who underwent resective operations, were seizure free for over 3 years and were no longer taking AEDs. For a better understanding of the beamformer performance, we selectively included magnetic resonance imaging (MRI) negative and lesional cases, typical mesial temporal and neocortical temporal cases, unilateral and bilateral dipole cases, as well as some invasive EEG implant cases. We retrospectively reviewed the results of presurgical MEG and scalp video EEG recordings. The patient characteristics are described in Table 1. The study had the approval of the Xuanwu Hospital Ethics Committee and was in accordance with the Declaration of Helsinki.

**Table 1:** Detailed demographics, clinical data, and MEG analysis results of the 14 patients studied.

**No**.	**Age range**	**Past history**	**MRI**	**Scalp EEG**				**Invasive EEG**		**SECD**	**Beamformer**	**Surgery**	**Pathology**
				**Interictal ED**	**Intermittent slow**	**Sz onset**	Scalp EEG lateralization^*^	**Location of ICE**	**Sz onset**				
1	25–30	–	Normal	Occasional, R	Bilateral, L = R	N	N	Bi-T	R	R temporal, insular	R	R tailored ATL	FCD
2	40–45	–	Normal	R	R	Bilateral, R>L	R	–		R temporal	R	R ATL	FCD, HS
3	15–20	–	Hyper T2 in L temporal cortex, atrophy in R hippocampus	Bilateral, L>R	L	Bilateral, L>R	N	Bi-T	L	L temporal	L	L tailored ATL	FCD
4	5–10	–	R HS	Bilateral, R>L	Bilateral, R>L	R	R	–		R temporal	R	R ATL	FCD, HS, gangliogliomas (WHO I)
5	10–15	FC	R HS	R	Bilateral, R>L	R	R	–		R temporal	R	R ATL	FCD, HS
6	25–30	FC, encephalitis	R HS	R	Bilateral, R>L	R	R	–		R temporal	L	R ATL	FCD, HS
7	15–20	–	L HS	L	Bilateral, L>R	L	L	–		L temporal	bilateral	L ATL	FCD HS
8	20–25	–	L HS, R arachnoid cyst in temporal pole	L	Bilateral, L>R	N	N	Bi-T	L	L temporal	L	L ATL	FCD, HS
9	40–45	–	Normal	Bilateral, R>L	Bilateral, L = R	R	N	Bi-T	R	L temporal, R parietal	R	R ATL	FCD
10	30–35	–	Normal	Bilateral, R>L	Bilateral, L = R	N	N	Bi-T	R	Bi-temporal	bilateral	R tailored ATL	FCD
11	20–25	Meningitis	Hyper T2 in R hippocampus	Bilateral, R>L	Bilateral, L = R	Bilateral, R>L	R	–		L frontal, R temporal	bilateral	R ATL	FCD, HS
12	35–40	–	bilateral HS	R	R	R	R	–		R temporal	R	R ATL	FCD, HS
13	30–35	FC	Atrophy in R hippocampus	R	R	R	R	–		R temporal	R	R ATL	FCD
14	15–20	FC	Hyper T2 in L	L	Bilateral, L = R	L	L	–		L temporal	L	L ATL	FCD, HS

ED, epileptiform discharges; MRI, magnetic resonance imaging; EEG, electroencephalogram; FC, febrile convulsion; SECD, single equivalent current dipole; FCD, focal cortical dysplasia; HS, hippocampal sclerosis; R, right; L, left; ATL, anterior temporal lobectomy; N, non-lateralizable; Bi-T, bilateral temporal; ICE, intracranial electrodes; Sz, seizure;

* *, lateralization based on comprehensive EEG analysis*.

### EEG recording

All patients had long-term continuous video EEG monitoring, which lasted 3–10 days depending on the number of seizures captured. The electrodes were placed according to the 10-20 International Electrode System, and bilateral sphenoidal electrodes were added. Referential and bipolar montages were used to investigate the interictal epileptiform discharges and slow waves. All of the scalp video EEG data were read by an experienced EEG technician (NH) during presurgical evaluation and were signed by a clinical epileptologist (LL). Both the technician and clinical epileptologist were blinded to the MEG results.

### MEG recording

Spontaneous MEG signals were recorded using a 306-channel, whole-head MEG system (VectorView™, Elekta Neuromag, Helsinki, Finland) with a sampling rate of 1000 Hz and filtering of 0.1–300 Hz for ~60 min. The system comprised 102 locations at triplets, including one magnetometer and two orthogonal planar gradiometers. A three-dimensional digitizer, a Polhemus™ system (Colchester, NH, USA), was used to determine the location based on anatomical fiducial points for the following MRI-MEG coregistration. During the recording, the patient laid supine, and immobile with their head positioned within a helmet-shaped sensor inside a magnetically shielded room. Any section of the recordings with non-cerebral artifacts (magnetic artifacts due to metal objects, strong cardiac signals, or environment noises) was removed.

### SECD model

The interictal epileptiform spikes were typically identified by visual screening of the recorded data by an experienced neurologist (XZ), and their sources were localized by the SECD method using Neuromag software (Elekta, Stockholm, Sweden) and coregistered to the patient's MRIs. The location, strength, and orientation of dipole sources that best fit the measured magnetic fields were calculated at the peak of the global field power of each spike. MEG spike sources with goodness of fit values >85% were considered significant. We classified SECD lateralization into three categories based on their distributions, according to “75% rules” (8). If all dipoles were distributed in one hemisphere or with a side-to-side ratio >3:1, we defined it as left or right unilaterality. If all dipoles were distributed bilaterally with a side-to-side ratio lower than 3:1, we defined it as bilaterality. We analyzed the same MEG spikes in SECD and spike segment for beamformer spikes.

### Beamformer model

The MEG head model was reconstructed using both Freesurfer and the MNE toolbox. Source space was simulated with an unconstrained-source grid based on the gray matter boundary obtained from each individual subject's T1-weighted MRI using Freesurfer (20, 21) and a grid spacing of 7 mm. The MEG sensors were carefully registered on the MRI-based head shape according to the digitized head points. The process of registration was conducted using the function mne_analyze in the MNE toolbox. Lead field vectors were calculated using the MNE toolbox (function: mne_do_forward_solution). A beamformer, as spatial filters, passed the desired signal at a specified source location while attenuating activity originating from other locations. In this study, linearly constrained minimum variance beamformer (22) was employed to extract source waveforms at each source location. The beamformer analysis was performed by an experimenter (JZ) blinded to patient clinical information. For each patient, beamformer analysis was performed on at least two segments with spikes and one segment without spikes (resting state). Each segment contained 9 s. The spike we chose to analyze had no neighboring artifacts or other spikes. To include more analyzable segments, we used a 9-s segment duration. The resting segments were “clean” segments without artifacts or spikes. The detailed steps to apply the beamformer method to analyze MEG recordings were as follows:

1. For each subject, several 9-s MEG segments, with or without spikes, were extracted.
2. Band filters were applied to each segment and thus, each segment generated 5 new filtered MEG segments. The filtered bands were set to 1–3, 4–7, 8–12, 13–29, 30–70, and 1–70 Hz, respectively. This step was conducted using the function mne_process_raw from the MNE toolbox.
3. A self-customized beamformer algorithm was employed, second by second, to each filtered segment, and we obtained each second source waveform at a specific frequency for each type of MEG segment (with or without spikes). This process was conducted in matlab using code that we wrote ourselves. The MNE toolbox provided easily used matlab based packages: function read_volume_source_space was used for reading individualized lead fields.
4. Based on (3), by averaging the absolutes of the 9-s source waveforms over time, one could obtain the averaged source power distribution at a specified frequency. The function mne_volume_data2mri was used to transform the beamformer-based source reconstruction into a format that could be displayed on the cortical surface as functional images in the function tkmedit (from Freesurfer).

Adding each source power within one hemisphere together, the power distribution of the beamformer sources could be easily calculated.

### Feature extraction from beamformer results

All thresholds used to display the power images in a cortical model based on Freesurfer reconstruction were set to 80% of the maximum power among all grid points. For each segment, we calculated the power distribution of beamformer sources (denoted as the lateralization index, LI) and averaged them to obtain the LI for each patient. The LI was calculated using the well-known basic formula (23):

$$\begin{array}{l} \text{LI}=\frac{{\text{Q}}_{\text{LH}}-{\text{Q}}_{\text{RH}}}{{\text{Q}}_{\text{LH}}+{\text{Q}}_{\text{RH}}} \end{array}$$

where Q represents the activation power in the appropriate hemisphere. Thus, the LI ranges from −1 to 1 to indicate right to left hemispheric dominance. We defined lateralization by beamformer based on LI at the delta frequency. The lateralization was left when the LI was >0.05, and the lateralization was right when the LI was <-0.05. LI values ranging from −0.05 to 0.05 defined bilaterality.

To explore the time dependency of the power distribution of beamformer sources, we measured the power with a 9-s duration and analyzed the power distribution at the point of each second.

To explore the frequency dependency of the power distribution of beamformer sources, we calculated the LI at multiple frequency bands, including delta (1–3 Hz), theta (4–7 Hz), alpha (8–12 Hz), beta (13–29 Hz), gamma (30–70 Hz), and full bands (1–70 Hz).

## Results

### Patient characteristics

Our study included 14 patients (7 men and 7 women). The age range was 6–41 years (Median = 25.5, IQR 18.25, 31.75), and the epilepsy duration was 3–20 years (Median = 12.5, IQR 9.5, 15.5). Four patients had a history of febrile convulsion, and one had encephalitis at 2 years of age (patient #6). Patient #11 had meningitis at 8 months old. The others had unremarkable past history. MRI abnormalities, in the forms of hippocampal sclerosis (HS), hippocampal atrophy, local hyper T2 signal and arachnoid cyst in the temporal lobe, were found in 10 patients. MRI images were normal in 4 patients. Five patients underwent bilateral temporal invasive EEG implantation. Eleven patients underwent unilateral standard anterior temporal lobectomy, and three underwent tailored temporal lobectomy. All patients were seizure free at more than 3 years follow-up. Histopathological investigations confirmed focal cortical dysplasia (FCD) in 5 patients, FCD associated with HS in 8 patients, and gangliogliomas associated with HS and FCD in one patient.

### SECD analysis

In all patients, the interictal spike sources were localized using SECD analysis, and the results are detailed in Table 1. Eleven patients had unilateral dipole clusters and underwent ipsilateral surgery. The remaining three patients had bilateral distributed dipole clusters (patient #9–11). Ten patients underwent complete resection of the dipole distribution. Four patients (patient #8–11) underwent incomplete resection of the dipole distribution.

### Beamformer analysis

#### Time-dependent power fluctuation

**Figure 1:**
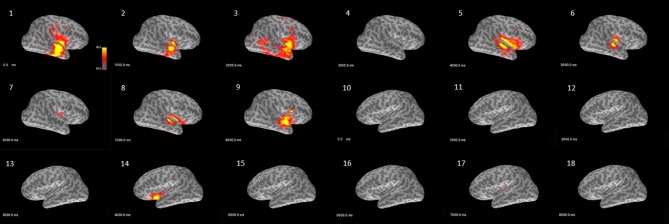
Example demonstrating the characteristic of power over time analyzed with the beamformer model (patient #1). This activity map shows the delta band power fluctuation in bilateral hemispheres across 9 s of a resting segment. The images (1–9) are from the right hemisphere and reveal that power waxed and waned over time in the temporal and perisylvian fissure area. The images (10–18) are from the left hemisphere and only show relatively weak power at the fifth second in the perisylvian fissure area.

The beamformer results showed that the power fluctuated with time, especially in the delta frequency band. Figure 1 shows an example of patient #1, with right temporal epileptogenicity. In the 9-seccond resting segment, the first, third, fifth and ninth seconds displayed a relatively high power distribution in the right temporal lobe. Most patients had similar time-dependent power fluctuations in the delta frequency band. Figure 1 was created using the function mne_make_movie from the MNE toolbox.

#### LI at multiple frequency bands

**Figure 2:**
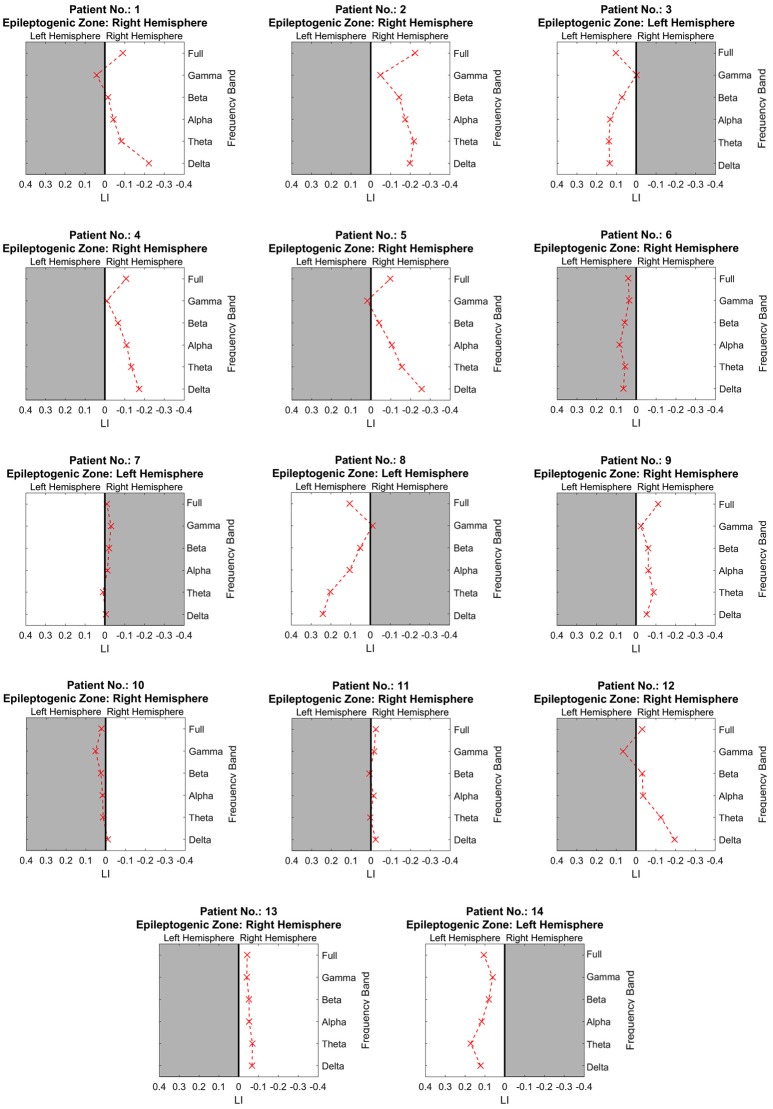
The lateralization accuracy of beamformer at all frequency bands for each patient. At each panel showing the results of one patient, the x coordinates denote the lateralization index, and the y coordinates represent different frequency bands. The crosses on the right side of the midline indicate a right-dominated power distribution, and those on the left indicate a left-dominated power distribution. The white background denotes lateralization correlating with the side of resection as determined at presurgical conference.

**Figure 3:**
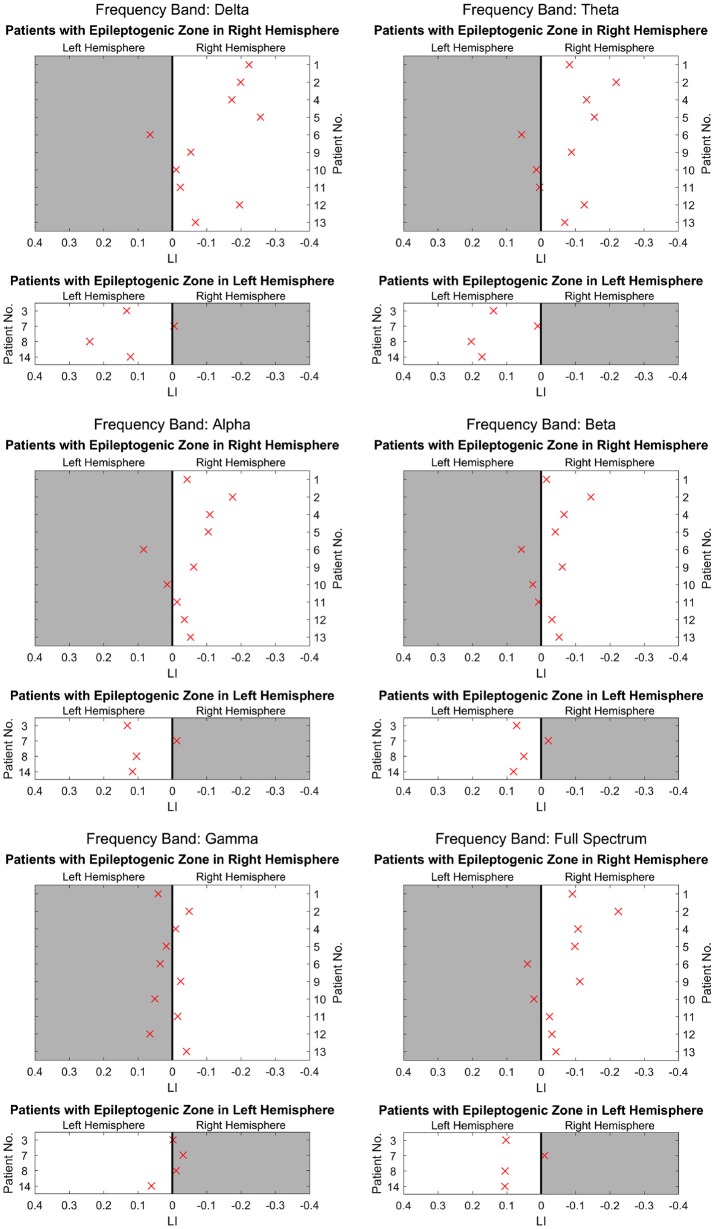
The lateralization accuracy of beamformer for all patients at different frequency bands. At each panel showing results at each frequency band, the x coordinates denote the LI, and the y coordinates represent different patients. The upper 10 patients had epileptogenic regions on the right hemisphere, and the lower 4 patients had these regions on the left. The crosses on the right side of the midline indicate a right-dominated power distribution, and those on the left indicate a left-dominated power distribution. The white background denotes lateralization correlating with the side of resection as determined at presurgical conference. The delta band has the most crosses on the white background, indicating high lateralization values. The theta, alpha, and beta bands have a majority of crosses on the white background, although they appear dispersed and near the midline. However, in the gamma band, crosses are distributed almost equally on the white background and gray background, indicating poor lateralization.

Figure 2 shows the separated LIs of 14 patients at different frequency bands. In the delta frequency band, 7 patients had right-dominant LIs. Four patients had left-dominant LIs. Of these 11 patients, 10 patients had a dominant power distribution ipsilateral to the resection. In the other three cases, the power distribution was bilateral. Different frequency bands had variable lateralization values. Figure 3 further demonstrates these differences among multiple frequency bands. With more strongly lateralized LI, the delta band showed the best lateralization value. The theta, alpha, and beta bands achieved fair lateralization values. The gamma band failed to lateralize.

#### LI and interictal spike

We analyzed at least two segments with spikes and one segment without spikes for each patient. In 10 patients whose LI results were concordant with resection, there was no difference between spike segments and non-spike segments.

### Comparison among SECD, beamformer, and EEG

Table 2 shows a comparison of scalp video EEG, SECD and beamformer analyses. The SECD results revealed that 11 patients had unilateral dipole clusters ipsilateral to resection. The beamformer results showed that 10 patients had a dominant power distribution ipsilateral to resection. Comparatively, 9/14 patients were lateralized correctly based on continuous video EEG. Fisher's exact test was used to compare the rate of lateralization between EEG and beamformer, SECD and beamformer, and EEG and SECD. No significant difference was found.

**Table 2:** Lateralization results for continuous video EEG, SECD, and beamformer analyses.

**No**.	**EEG**	**SECD**	**Beamformer**	**Resection (side)**
1	N	R	R	R
2	R	R	R	R
3	N	L	L	L
4	R	R	R	R
5	R	R	R	R
6	R	R	L	R
7	L	L	N	L
8	N	L	L	L
9	N	N	R	R
10	N	N	N	R
11	R	N	N	R
12	R	R	R	R
13	R	R	R	R
14	L	L	L	L

*EEG, electroencephalogram; SECD, single equivalent current dipole R, right; L, left; N, non-lateralizable*.

Further analysis was conducted based on the details of scalp video EEG in Table 1. In 5 patients with bilateral interictal epileptiform discharges on scalp EEG, beamformer lateralization correlated with resection in 3 patients and SECD in 2 patients. In 5 patients with implanted EEG, beamformer lateralization correlated with resection in 4 patients and SECD in 3 patients. In 10 patients who had intermittent slow activity in bilateral temporal regions, 5 had some lateralizing signs (prominent on one side) on scalp EEG. For the other 5 patients who did not have definite lateralizing signs on scalp EEG, beamformer lateralization correlated with resection in 3 patients, and SECD in 2 patients.

## Discussion

In presurgical evaluation for TLE, accurate lateralization is very important especially when bilateral temporal epileptiform discharges or structural abnormalities are present. Several neuroimaging modalities have been investigated in lateralization in TLE, such as proton magnetic resonance spectroscopy (^1^H-MRS) and diffusion tensor imaging (DTI). MRS has the potential to detect metabolic abnormalities in the temporal lobe non-invasively. In MRI-negative TLE cases, the ^1^H-MRS using an asymmetry index provided accurate lateralization in 87% of the patients (24), or had a 60–75% concordance with EEG or intracranial EEG findings (25). In patients with positive MRI findings, ^1^H-MRS provided accurate lateralization in 57% of the patients (26) and indicated 100% concordant lateralization with EEG findings in unilateral TLE and 75% concordance with intracranial EEG findings in bilateral TLE (25). DTI is able to indirectly evaluate structural integrity by providing information about the direction and magnitude of water diffusion. In patients with positive MRI findings, DTI lateralized accurately in 57% of the patients (26). Neither of these neuroimaging modalities alone achieved satisfactory lateralization. Our study evaluated the lateralization values of beamformer in 14 TLE patients, with the expectation that the beamformer could provide complementary information for lateralization.

### Beamformer's performance in lateralization

A variety of mathematical methods are clinically used to model epileptiform discharge location and topography with MEG. As a classic method, SECD analysis is easy to learn and has been commonly used in presurgical evaluation but has some limitations. First, SECD analysis is effective for small focal sources, but it does not accurately characterize extended sources. In those cases with extended sources, SECD location may simply pinpoint the geometric center of the active cortex (27). Second, SECD analysis is performed on data segments containing an adequate number of spikes (one dipole per spike). If few spikes are recorded or the localization of dipoles is scattered, then the results are questionable. Beamformer analysis is a promising method because, as an algorithm using spatial filtering, it does not suffer from background noise in the source localization calculation (28). However, as beamformer is a time-consuming method, it is necessary to evaluate this approach in presurgical workup.

Our study showed that beamformer lateralized correctly in 10/14 patients. SECD had comparable lateralization values. A combination of the two analysis methods might increase lateralization value. SECD analysis is necessarily dependent on spike pick up. However, for some patients, few spikes are captured in MEG. Notably, our study found that beamformer analysis could be performed based on MEG data without visible spikes and is able to lateralize as well. This finding has considerable practical significance. Although the lateralization accuracies of SECD and beamformer were comparable, in some patients, one of the methods correctly lateralized, whereas the other method failed to do so. Therefore, due to the complementary values of these two methods, a comprehensive analysis using both methods is necessary.

A comparison of three MEG algorithms, namely, SECD, current density reconstructions, and beamformer, was carried out by Tenney et al in 32 children with intractable epilepsy (29). They found that all algorithms have similar rates of concordance with intracranial EEG. This result was consistent with our findings. Additionally, they investigated the combinations of source algorithms as well but did not find significant differences in accuracy, positive predictive value, or negative predictive value. Tenney focused on localization on lobar or sublobar level and included not only temporal cases but also extra-temporal cases. Differently, our study mainly focused on lateralization on a hemisphere level in temporal cases. Moreover, in our study, a linearly constrained minimum variance beamformer was used, and voxel waveforms were extracted. In contrast, in Tenney's study, synthetic aperture magnetometry with excess kurtosis was used. Hall et al retrospectively applied kurtosis beamforming analysis in 22 heterogeneous epilepsy patients and compared it with SECD results which previously had been analyzed in presurgical evaluation (30). The kurtosis beamformer overlapped with the resection cavity in 9/13 seizure free patients, and SECD in 10/13. The sublobar accuracy of the kurtosis beamformer with respect to the resection zone was higher than ECD (56 and 50%, respectively) on a sublobar level although the differences were not statistically significant. They suggested kurtosis beamforming may provide additional value to SECD and should be integrated with existing clinical protocols. However, the dependence on spikes is still a general limitation of both kurtosis beamforming and SECD analysis. We found that linearly constrained minimum variance beamformer used in our study could analyze both of spike segments and non-spike segments, which provides an alternative method for cases with low spike frequency.

### MEG analysis vs. EEG

All patients had long-term continuous video EEG monitoring. Despite comprehensive analysis of video EEG, 5 patients did not have definite lateralization results and needed further invasive EEG implantation. In these 5 patients, beamformer lateralization correlated with the side of resection in 4 patients, invasive EEG in 5 patients and SECD in 3 patients. Therefore, 4 patients benefited from the use of beamformer in our study. Although invasive EEG achieved better lateralization than the beamformer, the EEG implantation has potential risk as an invasive technique, and the high cost of electrodes and related surgical procedures inevitably brings a heavy burden. Thus, beamformer might be a valuable technique that provides additional lateralization information for those cases in which lateralization is difficult by scalp EEG alone. We also compared video EEG with these two MEG analyses in Table 2, and no significant differences were observed. When all three techniques were considered, 13/14 patients could be lateralized correlating with the side of resection. One patient could only be lateralized by invasive EEG (Patient #10). Therefore, we suggest a combination of beamformer, SECD and scalp EEG in TLE as non-invasive evaluation tools, although none of these techniques can replace invasive EEG. Holmes et al. noted that the concordance of non-invasive factors with invasive EEG findings was more important than specific methods of intracranial recording or the magnitude of preponderance of ictal onsets to one side alone in predicting outcome (8). In a word, a combination of multiple presurgical evaluation techniques is clinically valuable, and concordance of these techniques may indicate better surgical outcome.

Patient #10 had false lateralization by beamformer analysis and non-lateralization by EEG analysis and SECD analysis. It was difficult to lateralize from 5 seizures with scalp EEG recordings. Following implantation of intracranial electrodes, 9 seizures were captured, and all arose from the right temporal lobe, with bilateral interictal spikes. This was the only falsely lateralized case for beamformer; however, EEG and SECD did not have any false lateralization. This false-positive finding might be due to undersampling in time with beamformer. A prolonged MEG might help overcome this disadvantage, but it is an inevitable disadvantage compared with continuous EEG. Beamformer analysis of more segments might also help to some extent, although it is limited to 1-h data. For example, if a shorter duration (<9 s) was chosen, more segments would be enrolled. The optimization of beamformer analysis warrants study in the future.

### Low frequency activity in beamformer

Our study suggested that the lateralization was best in low frequency band beamformer analysis. To understand whether the low frequency activity in beamformer is the same as intermittent slow activity in EEG, we reviewed intermittent slow activity on EEG for all patients. In 10 patients who had intermittent slow activity in bilateral temporal regions, 5 had some lateralizing signs (higher amplitude or percentage on one side) on scalp EEG. For the other 5 patients who did not have definite lateralizing signs on scalp EEG, beamformer lateralization correlated with the side of resection in 3 patients and SECD in 2 patients. Therefore, the low frequency power fluctuation found on beamformer analysis might provide us additional lateralizing information to scalp EEG.

This slow activity found in MEG could be a marker of regional dysfunction and structural damage. Some researchers have discussed the relationship between slow activity and epileptogenicity. Ishibashi et al. (27) observed lateralized focal low frequency magnetic activity on the side ipsilateral to the epileptogenic region and a maximum amplitude in mesial TLE with HS. In another study (31), researchers calculated the slow wave dipole density in the frequency range of 2–6 Hz and found that the slow activity was produced within an area at the border of the spike generation area and was indeed more extended. This area might contain tissue damaged by epileptogenic processes and hence produce pathological slow activity. This activity was probably related to a chronic functional deactivation of the lateral temporal neocortex due to a disruption in thalamocortical and corticolimbic projections (27). Some human electrocorticography studies indicated that cortical slow waves represent inhibition of neuronal firing (32). Serafini and Loeb (33) studied subdural grid EEG recordings from 10 epilepsy patients, measured the amplitudes of slow waves and of sharp waves and compared their ratios in all the electrodes where discharges were involved. They found that the slow-wave/sharp-wave ratio increases several folds within 2–3 cm from the highest sharp wave electrode. The initial sharp wave is thought to reflect excitatory postsynaptic potential synchronization and the slow afterwave is thought to reflect subsequent inhibition (32). Therefore, the prevalence of slow waves at the periphery of epileptogenic foci probably indicated surrounding enhanced inhibition, which limited the spread of epileptic activity. The above-mentioned studies all support the important lateralization value of low frequency MEG, consistent with our study. In the future, further research might focus on the slow activity in patients with epilepsy.

We analyzed both segments with spikes and without spikes using beamformer based on low frequency MEG signal after filtering and found that the low frequency activity waxed and waned continuously with a cycle of 2–3 s (~0.3–0.5 Hz) rather than maintaining a constant state, irrespective of the presence or absence of visible spikes in 1-h data. This result suggested that there might be a regional continuous power fluctuation on the epileptogenic side even during the resting state. As the fluctuation was mainly located around the epileptogenic region, it was more likely to be pathological and epileptogenic. fMRI research found that infra-slow EEG fluctuations were correlated with resting state network dynamics (34). One remaining question is whether the disturbed low frequency activity fluctuation in resting state network dynamics gave rise to the process of epilepsy. An improved understanding of the properties of low frequency power oscillation in epilepsy might provide further clues pertaining to epileptogenesis.

### Bilateral temporal spikes in unilateral temporal epilepsy

Patients with bilateral temporal spikes constitute a significant portion of all patients with temporal epilepsy. In our study, there were 5 cases with bilateral spikes on scalp EEG analysis, 3 cases with bilateral dipoles on SECD analysis and 3 cases with bilateral involvement on beamformer analysis. The percentage was consistent with previous studies.

Bilateral spikes probably represent an extended epileptogenic region. Morrel hypothesized that these spikes were a sign of the progressive nature of epileptogenesis by using a rat model (3). However, in these “bilateral cases,” seizures usually originate from one area only, as observed in the 14 patients in our study. Moreover, contralateral spikes usually disappeared after the resection of the primary epileptogenic focus, which indicated that bilateral temporal spikes did not automatically indicate a bilateral epileptogenicity (1, 35–37).

Frequent contralateral seizure propagation is associated with the presence of bilateral temporal spikes (38). Gotman and Koffler found that spikes are primarily influenced by the preceding seizures (39). Jansjky et al. also found that the degree of the lateralization of epileptiform discharges mainly depends on whether the preceding seizure involved one hemisphere or both sides (40). Because all patients in our study became seizure free after anterior temporal lobectomy, we considered that the presence of bilateral temporal spikes may predominantly be a result of the seizure activity involving both hemispheres. Additionally, during presurgical evaluations, AEDs were usually reduced or withdrawn. This might inevitably have influenced the increase in bilateral spikes.

### Limitations

This study has several limitations. First, it was conducted with a relatively limited number of patients. Our findings preliminarily verified the potential lateralization value of beamformer, but caution is needed when generalizing the results of this study. Second, non-seizure-free cases might have different etiologies, and uncontrolled variants would interfere with the analysis in a study with such a small sample size. Therefore, the patients presented in this study were a selected group (all seizure-free). The beamformer performance in non-seizure-free cases remains unclear and needs to be explored in the future. Third, this study was retrospective, and we therefore could not control the quality of MEG data. Some patients in our study had many artifacts in their MEG data, which made it difficult to find a segment longer than 9 s. Last but not least, the surgery strategy was determined based on semiology, MRI, continuous video EEG, and SECD analysis at presurgical conference. Therefore, the resection side was determined in part by the variables under study (continuous video EEG and SECD analysis) and was probably more consistent with continuous video EEG and SECD analysis compared with beamformer analysis, which was not considered when the resection plan was made.

## Conclusion

In summary, our results supported the potential lateralization value of beamformer analysis as an adjunctive method in presurgical workup. Beamformer may provide additional value especially when spikes are not clearly conclusive on the sensors or when dipoles are scattered. The different detection sensitivities make beamformer, SECD and scalp EEG complementary, therefore we suggest using all the methods in clinical practices. The delta frequency band had a higher lateralization value than other frequency bands. Slow wave activity analysis seems to be a promising contribution that may help define the epileptogenic region in the future. We believe that slow wave activity analysis will advance our knowledge of epilepsy presurgical strategies and epileptogenesis.

## Author contributions

YL and YW contributed at all stages of manuscript preparation. YY, YZ, and ZH acquired the clinical and MEG data. XZ performed SECD analysis. JZ and ZZ performed beamformer analysis and made figures. YL designed the study, oversaw data acquisition and wrote the manuscript. JZ and YW analyzed the MEG data and critically revised the manuscript. LL and NH acquired, analyzed and interpreted the EEG data and critically revised the manuscript.

### Conflict of interest statement

The authors declare that the research was conducted in the absence of any commercial or financial relationships that could be construed as a potential conflict of interest.
